# Oxidative status of maternal blood in pregnancies burdened by inherited thrombophilias

**DOI:** 10.1371/journal.pone.0234253

**Published:** 2020-06-17

**Authors:** Jelena Bogdanović Pristov, Miloš Opačić, Milica Bajčetić, Vesna Mandić, Dragana Maglić, Željko Miković, Ivan Spasojević

**Affiliations:** 1 Life Sciences Department, Institute for Multidisciplinary Research, University of Belgrade, Belgrade, Serbia; 2 Department of Pharmacology, Clinical Pharmacology and Toxicology, School of Medicine, University of Belgrade, Belgrade, Serbia; 3 Clinical Pharmacology Unit, University Children's Hospital, Belgrade, Serbia; 4 Department for High-risk Pregnancies, University Clinic for Gynecology and Obstetrics “Narodni front”, Belgrade, Serbia; 5 Faculty of Medicine, University of Belgrade, Belgrade, Serbia; Universita degli Studi di Milano-Bicocca Scuola di Medicina e Chirurgia, ITALY

## Abstract

Oxidative status of maternal blood represents an important parameter of pregnancy that is involved in both, regulation of physiological processes and (if significantly altered) development of different pregnancy complications. Inherited thrombophilias represent genetic disorders that increase the risk of thromboembolism in pregnancy. Little is known about the impact of thrombophilia on the oxidative status of maternal blood. In this study, we analyzed oxidative status of blood of 56 women with pregnancies burdened by inherited thrombophilias. The status was established at three different trimesters using biochemical assays and electrochemical measurements, and it was compared to 10 age- and trimester-matching controls. Activities of superoxide dismutase, catalase, and glutathione reductase in the 1^st^ and the 2^nd^ trimester of thrombophilic pregnancy were lower than controls. Also, there was less oxidation in the plasma, according to higher concentration of reduced thiols and lower oxidation-reduction potential. Therefore, it appears that thrombophilic mothers do not experience oxidative stress in the circulation in the first two trimesters. However, the rise in GPx, GR and SOD activities in the 3^rd^ trimester of thrombophilic pregnancy implies that the risk of oxidative stress is increased during the late pregnancy. These results are important for developing antioxidative treatment that could tackle thrombophilia-related pregnancy complications.

## Introduction

Oxidative status of maternal blood is an important parameter of pregnancy [1]. It has a regulatory role in the processes of placental angiogenesis and development, gestation maintenance, and the recognition of fetus-cells and immune maternal responses [2–4]. A positive correlation has been established between oxidative status of the mother and the neonate [5]. It is important to note that several studies have found that normal pregnancy is characterized by mild pro-oxidative changes in the status of maternal blood when compared to non-pregnant or postpartum women [6–8]. However, more pronounced pro-oxidative changes (*i*.*e*. oxidative stress) have been related to the development of different pregnancy complications, such as pre-eclampsia, fetal growth restriction, miscarriage, and others [1, 9, 10]. Further, the development of oxidative stress in the blood is known to induce thrombogenesis via mechanisms that have been extensively studied [11–13], and which appear to take place in pregnancy complications that are related to oxidative stress [14, 15]. However, whether pro-thrombic conditions that are induced by some other factors, such as inherited thrombophilias, may lead to oxidative stress, is not clear.

Inherited thrombophilias represent genetic disorders that increase the risk of different complications, fetal loss, and thromboembolism in pregnancy [16–18]. It has been suggested that more than half of vascular complications of pregnancy can be attributed to genetic thrombophilias [19]. Pro-thrombic state results in haemostatic response and microthrombi formation [17]. This may further lead to increased production of reactive oxygen species in the circulation in relation to ischemia, mechanical stress/endothelial injury, and/or inflammation [20, 21]. Pertinent to this, we have found previously that oxidative status of thrombophilic mothers shows significant pro-oxidative changes postpartum, immediately following the delivery [22, 23]. Besides this, little is known about the impact of thrombophilia on the oxidative status.

The aim of the present study was to determine oxidative status of maternal blood during pregnancy burdened by inherited thrombophilias, and to compare it to women with normal pregnancies. It is important to note that pregnancy represents a dynamic period with maternal circulation undergoing significant physiological changes to meet the demands of the fetus and the mother. For example, cardiac output rises by increasing heart rate and stroke volume, reaching ~50% above the pre-pregnancy baseline near the end of the pregnancy [24, 25]. Also, it appears that oxidative status of maternal blood shows a timeline of changes during normal pregnancy [26, 27]. Therefore, it was important to measure parameters of oxidative status at different timepoints–trimesters. Knowing oxidative status at different trimesters may be particularly important for improving the performance of antioxidant supplements in pregnancy by matching the time of application with the periods of high risk of oxidative stress development. The best approach in assessing oxidative status of circulation is to analyze: (i) antioxidative enzymes in erythrocytes: superoxide dismutase (SOD; converts intracellular superoxide radical anion to hydrogen peroxide (H_2_O_2_)), and catalase (CAT), and glutathione peroxidase (GPx) and glutathione reductase (GR) couple, which remove H_2_O_2_ [28–31]; and (ii) redox parameters of plasma: the level of reduced thiols (R-SH; highly susceptible to oxidation) [32], and static oxidation-reduction potential (ORP; an integrated comprehensive metabolomic analyte that measures the balance between stable oxidative and reductive species in biological fluids) [33, 34].

## Materials and methods

### Patients

Study cohorts included 56 pregnant women with inherited thrombophilias, and three groups of 10 age- and parity-matching women with normal pregnancy–controls (population details are available in Table 1).

**Table 1:** Population characteristics.

	Controls					Thrombophilia	Types of thrombophilia	n	Zygosity^#^
	1^st^ trimester	2^nd^ trimester			3^rd^ trimester				
n	30					56	Protein S deficiency	4	-
	10		10	10					
Age (years)*	35.4 ± 1.0					33.0 ± 0.7	*F2* gene mutation	6	1/5
	35.4 ± 1.5		33.7 ± 1.7	37.0 ± 1.9					
Parity*	0.96 ± 0.13					0.58 ± 0.10	Factor V Leiden mutation	13	1/12
	0.90 ± 0.23		1.20 ± 0.20	0.80 ± 0.20					
Chronic hypertens. (n)	0					6	*PAI-1* gene polymorphism	31	20/11
Gestational hypertens. (n)	0					14	*MTHFR* gene mutation	2	1/1
Pre-eclampsia (n)	0					8			

* Values are presented as mean ± standard error. Values for controls are presented for all 30 subjects, and for 10 subjects in each trimester.

^#^ Presented as homozygotes (n) /heterozygotes (n)

Inherited thrombophilias were detected by evaluating the presence of specific mutations/deficiencies: protein S deficiency, prothrombin *i*.*e*. coagulation factor II (*F2*) gene mutation, Factor V Leiden mutation, plasminogen activator inhibitor-1 (*PAI*-1) gene polymorphism, and methylenetetrahydrofolate reductase (*MTHFR*) gene mutation [35, 36]. All patients were diagnosed before the index pregnancy. Other complications involved: chronic hypertension—blood pressure exceeding 140/90 mm Hg before pregnancy or before gestation week 20, gestational hypertension–hypertension that developed after gestation week 20, and pre-eclampsia. All women with thrombophilia received therapeutic doses of low molecular weight heparin, from the beginning of the pregnancy to the postpartum period. Normal pregnancies were eligible for the control group if they had an uncomplicated pregnancy that resulted in the birth of a healthy newborn and no history of thromboembolism. No smokers were involved in the study. All patients (controls and thrombophilic) received 1000 mg of vitamin C and 5 mg of folate each day until gestational week 16; after that all patients received multivitamin supplement that contained 100 mg of vitamin C each day. Institutional approval for the study was granted by The Ethics Committee of University Clinic for Gynecology and Obstetrics „Narodni front“, in accordance with internationally accepted ethical standards (The Helsinki Declaration of 1964, as revised in 1975, 1983 and 1989), and each patient had signed the informed consent form.

### Samples

Blood samples (3 mL) were taken from all 56 pregnant women with thrombophilia in the 1^st^ (gestational week: 10–12), the 2^nd^ (gestational weeks: 22–26), and the 3^rd^ trimester (gestational week: 34–38). Control samples were collected from 10 women with normal pregnancy for each trimester in the same periods of gestation. The blood extraction was performed at the same time to routine clinical measurements. Venous blood was collected after overnight fasting using tubes containing 0.072 mL of 7.5% K_3_EDTA as the anticoagulant (Vacuette EDTA, Greiner Bio-One, Austria), and centrifuged at 2000 g/15 min/4°C to separate erythrocytes and plasma. Erythrocytes were washed three times with 0.9% NaCl at 4°C. Plasma and washed erythrocytes were immediately placed in tubes, snap-frozen in liquid nitrogen, and stored at -80°C for further analysis.

### Biochemical and electrochemical analysis

All chemicals were purchased from Merck (Darmstadt, Germany). Erythrocytes (0.5 mL) were lysed by adding 3 mL of ice-cold distilled water. Hemoglobin (Hb) concentration was measured by Drabkin method. SOD activity was determined by the adrenaline method [37]. One unit of activity is defined as the amount of enzyme that decreases the rate of adrenaline auto-oxidation at pH 10.2 by 50%. Interference with Hb was eliminated by precipitation prior to the assay using ethanol/chloroform (1:1, v/v), that was followed by centrifugation at 5000 g/5 min/4°C. The activity of CAT was determined as described previously [38]. One unit is defined as the amount of enzyme that reduces 1 mM of H_2_O_2_ per min. SOD and CAT activities were normalized to Hb. The activity of GPx was determined using *t*-butylhydroperoxide as substrate [39], and expressed in μmol NADPH/min/g Hb. GR activity was assayed as reported by Glatzle *et al*. [40], and expressed in μmol NADPH/min/g Hb. The content of R-SH in plasma was determined according to Ellman [41]. Static ORP (in mV) was measured at room temperature using RedoxSYS System (Aytu BioScience, Inc., Englewood, CO, USA) [34]. Plasma (40 μL) was applied to a pre-inserted sensor, and the readings were initiated automatically.

### Statistical analysis

Statistical analyses were performed using STATISTICA 8.0 (StatSoft Inc., Tulsa, OK, USA). Results are presented as box. Boxes represent the median and the 25^th^ and 75^th^ percentiles; whiskers represent the non-outlier range. Outliers and extremes are defined as data point values that are more than 1.5× and 3× interquartile range outside the box, respectively. We analyzed differences between controls and thrombophilic pregnancies, controls and 4 different inherited thrombophilias taken separately (data for *MTHFR* gene mutation thrombophilia (n = 2) were an exception), controls and thrombophilic women with hypertension (n = 20), and thrombophilic women with and without hypertension using nonparametric two-tailed Mann–Whitney *U* test. The differences between different trimesters in thrombophilic pregnancies and between different types of inherited thrombophilia were established using Kruskal-Wallis ANOVA by ranks with Dunn’s *post hoc* test. Results were considered to be statistically significant if p < 0.05.

## Results

**Fig 1:**
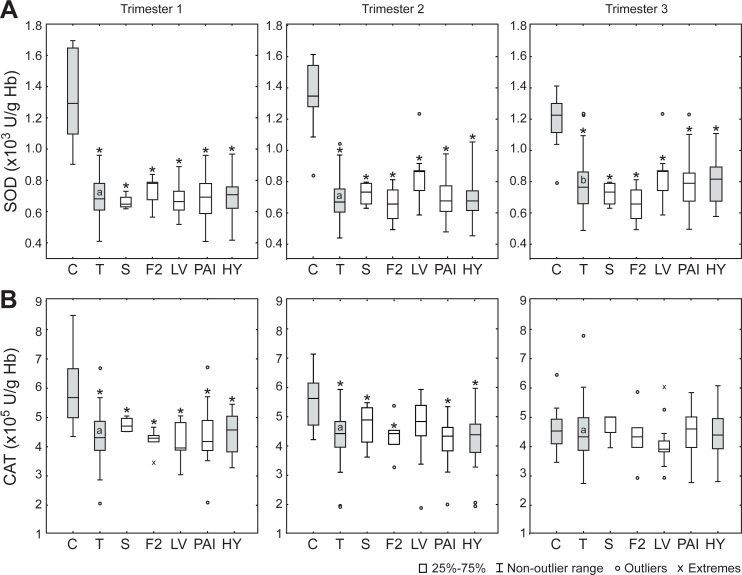
The activities of superoxide dismutase (SOD) and catalase (CAT) in erythrocytes in different trimesters. (**A**) SOD activity. (**B**) CAT activity. C–control (n = 10 for each trimester); T–thrombophilias (all; n = 56); S–protein S deficiency (n = 4); F2 –*F2* gene mutation (n = 6); LV–Factor V Leiden mutation (n = 13); PAI–*PAI*-1 polymorphism (n = 31); HY–thrombophilic pregnancies burdened with hypertension (n = 20). Boxes represent the median and the 25^th^ and 75^th^ percentiles; whiskers represent the non-outlier range; circles–outliers; x—extremes. *—Statistically different compared to control (p < 0.05). Different trimesters in thrombophilic pregnancy not sharing a common letter in the box are significantly different.

The activity of SOD in erythrocytes was about two-fold lower in thrombophilias than controls during the entire pregnancy (Fig 1A). Similarly, CAT activity was lower in inherited thrombophilias during the first two trimesters (Fig 1B). It is important to note that SOD activity in the 3^rd^ trimester of thrombophilic pregnancy was significantly higher than in the first two trimesters.

**Fig 2:**
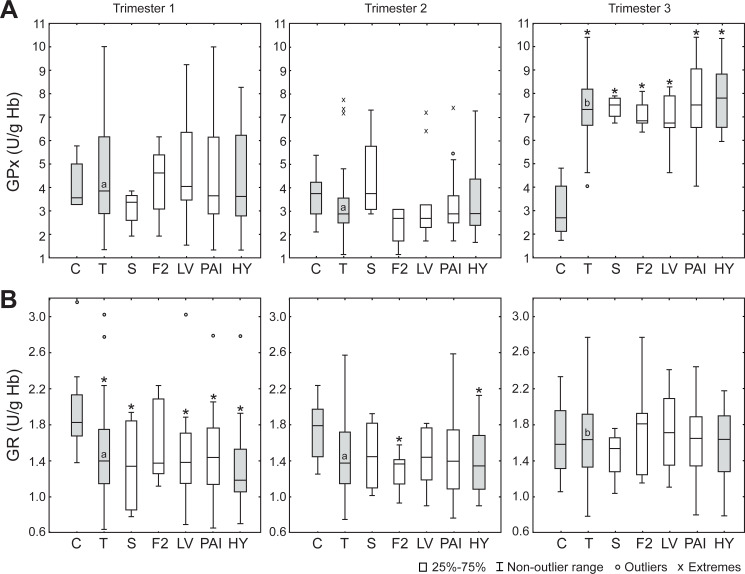
The activities of glutathione peroxidase (GPx) and reductase (GR) in erythrocytes in different trimesters. (**A**) GPx activity. (**B**) GR activity. C–control (n = 10 for each trimester); T–thrombophilias (all; n = 56); S–protein S deficiency (n = 4); F2 –*F2* gene mutation (n = 6); LV–Factor V Leiden mutation (n = 13); PAI–*PAI*-1 polymorphism (n = 31); HY–thrombophilic pregnancies burdened with hypertension (n = 20). Boxes represent the median and the 25^th^ and 75^th^ percentiles; whiskers represent the non-outlier range; circles–outliers; x—extremes. *—Statistically different compared to control (p < 0.05). Different trimesters in thrombophilic pregnancy not sharing a common letter in the box are significantly different.

Further, GPx activities in healthy and thrombophilic pregnancies were not significantly different in the first two trimesters (Fig 2A). In the 3^rd^ trimester, GPx activity showed a drastic increase in thrombophilic pregnancy and it was three-fold higher than in controls. GR activity was lower in inherited thrombophilias than controls only in the 1^st^ trimester (Fig 2B). Both, GPx and GR activities showed a significant increase in the 3^rd^ trimester of thrombophilic pregnancies compared to the first two trimesters.

**Fig 3:**
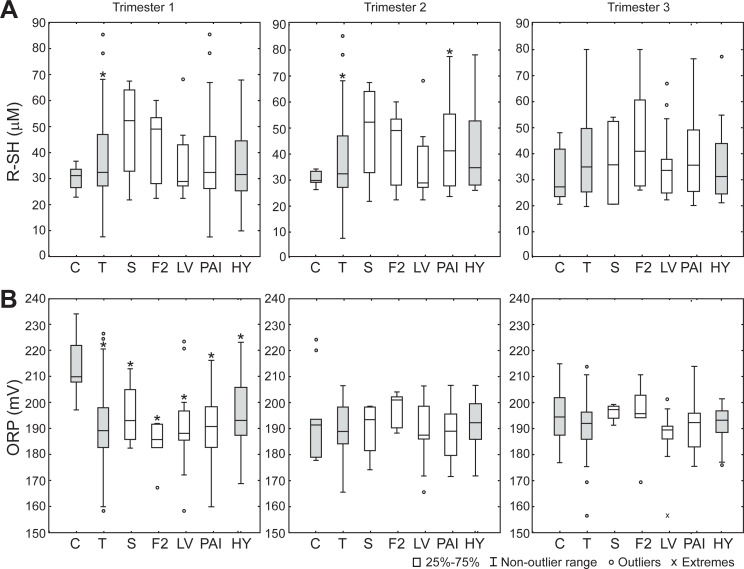
Redox settings in the plasma in different trimesters. (**A**) Concentration of reduced thiols. (**B**) Static ORP of plasma. C–control (n = 10 for each trimester); T–thrombophilias (all; n = 56); S–protein S deficiency (n = 4); F2 –*F2* gene mutation (n = 6); LV–Factor V Leiden mutation (n = 13); PAI–*PAI*-1 polymorphism (n = 31); HY–thrombophilic pregnancies burdened with hypertension (n = 20). Boxes represent the median and the 25^th^ and 75^th^ percentiles; whiskers represent the non-outlier range; circles–outliers; x—extremes. *—Statistically different compared to control (p < 0.05). There was no statistical difference between values obtained at different trimesters of thrombophilic pregnancy.

Finally, R-SH concentrations were higher in the 1^st^ and the 2^nd^ trimester, whereas ORP was lower in the 1^st^ trimester in the plasma of pregnant women with thrombophilia than controls (Fig 3). It is important to note here that lower ORP values stand for lower level of oxidizing and/or higher level of reducing species. So, the results implicate that there is less oxidation in thrombophilic pregnancies than controls. It is noteworthy that there were no changes in R-SH concentration and ORP in different trimesters of thrombophilic pregnancy. Further, there was no significant difference in any of the measured parameters between different types of inherited thrombophilia. Also, thrombophilias with hypertension showed similar trends as all thrombophilias in comparison to control values. There were no significant differences in any of the parameters between thrombophilic pregnancies with or without hypertension (not shown).

## Discussion

Surprisingly, the blood of pregnant women with inherited thrombophilias showed less oxidation than controls during the first two trimesters. Pro-reductive oxidative status is implicated by higher R-SH levels and by lower ORP in plasma, as well as by lower activities of SOD, CAT and GR in erythrocytes. Therefore, it can be concluded that thrombophilic mothers do not experience oxidative stress in the circulation in the first two trimesters. However, the rise in GPx, GR and SOD activities in the 3^rd^ trimester of thrombophilic pregnancy implies that the risk of development of oxidative stress is increased during the late pregnancy. This is in accord with our previous findings that placental tissue of thrombophilic mothers shows several-fold higher activity of H_2_O_2_-removing enzymes (CAT, GPx and GR) than controls [22], and that pro-oxidative changes of oxidative status take place in the blood of thrombophilic mothers immediately after the delivery [23]. It is possible that an increase in the cardiovascular load during pregnancy [24, 25], in combination with pro-thrombic conditions progressively leads to pro-oxidative pressure that exceeds the capacities of baseline antioxidative defense and provokes upregulation of antioxidative enzymes. Pertinent to this, GPx showed particularly pronounced increase near the term of thrombophilic pregnancy. Within erythrocytes, GPx appears to be mainly located close to the membrane. It has been proposed that GPx is in charge of preventing lipid peroxidation and removing H_2_O_2_ that enters the erythrocytes from plasma [42, 43]. Therefore, erythrocytes, in addition to placental antioxidative system [22], may play an important role in the protection of maternal circulation from excessive amounts of H_2_O_2_ in the last trimester of thrombophilic pregnancy. In close, it appears that near the term, pro-thrombic conditions lead to increased production of H_2_O_2_ in the circulation (in relation to ischemia/re-perfusion, endothelial injury, or inflammation [20, 21]). This is compensated by upregulated enzymatic antioxidative systems in erythrocytes and placenta. Erythrocytes have been proposed to act as a sink for extracellular H_2_O_2_ [44], whereas placental blood flow shows a large volume of 600–700 mL/min [45]. It is important to stress out here that H_2_O_2_ diffusion across cell membranes is facilitated by specific aquaporins (AQP) [46], which are present in placenta (AQP3, AQP8, AQP9) [47], and on erythrocytes membrane (AQP3) [48]. At the delivery, placenta is discarded and the protection of maternal circulation is weakened. So a pro-oxidative shift in oxidative status of blood takes place [22], which may be the cause of the increased risk of thrombosis and thromboembolism postpartum [49]. It is worth mentioning that no significant differences were observed between different thrombophilia types or between thrombophilic mothers with and without hypertension for any of the investigated parameters. This implies that the established trimestral profile of oxidative status is not related to a specific mutation or polymorphism or to the development of hypertension and that it represents a feature of thrombophilia *per se*. Finally, our results imply that the application of antioxidative supplements/therapy is most appropriate in the 3^rd^ trimester of thrombophilic pregnancies.

## Supporting information

S1 Dataset Data underlying the findings described are available in S1 Dataset. (XLSX)
